# Automatic Life Detection Based on Efficient Features of Ground-Penetrating Rescue Radar Signals

**DOI:** 10.3390/s23156771

**Published:** 2023-07-28

**Authors:** Di Shi, Gunnar Gidion, Leonhard M. Reindl, Stefan J. Rupitsch

**Affiliations:** Laboratory for Electrical Instrumentation and Embedded Systems, Department of Microsystems Engineering—IMTEK, University of Freiburg, Georges-Köhler-Allee 106, 79110 Freiburg, Germany; gunnar.gidion@imtek.uni-freiburg.de (G.G.); reindl@imtek.uni-freiburg.de (L.M.R.); stefan.rupitsch@imtek.uni-freiburg.de (S.J.R.)

**Keywords:** rescue radar, life detection, binary classification, feature engineering, one-way analysis of variance (ANOVA), minimum redundancy maximum relevance (MRMR), ground penetrating radar (GPR), respiratory signal, support vector machine (SVM)

## Abstract

Good feature engineering is a prerequisite for accurate classification, especially in challenging scenarios such as detecting the breathing of living persons trapped under building rubble using bioradar. Unlike monitoring patients’ breathing through the air, the measuring conditions of a rescue bioradar are very complex. The ultimate goal of search and rescue is to determine the presence of a living person, which requires extracting representative features that can distinguish measurements with the presence of a person and without. To address this challenge, we conducted a bioradar test scenario under laboratory conditions and decomposed the radar signal into different range intervals to derive multiple virtual scenes from the real one. We then extracted physical and statistical quantitative features that represent a measurement, aiming to find those features that are robust to the complexity of rescue-radar measuring conditions, including different rubble sites, breathing rates, signal strengths, and short-duration disturbances. To this end, we utilized two methods, Analysis of Variance (ANOVA), and Minimum Redundancy Maximum Relevance (MRMR), to analyze the significance of the extracted features. We then trained the classification model using a linear kernel support vector machine (SVM). As the main result of this work, we identified an optimal feature set of four features based on the feature ranking and the improvement in the classification accuracy of the SVM model. These four features are related to four different physical quantities and independent from different rubble sites.

## 1. Introduction

In disasters like earthquakes, people trapped under rubble may survive if rescued in time. Therefore, it is critical to deploy all possible urban search and rescue forces quickly. While rescue dogs are highly effective in finding victims due to their superior sense of smell, they can be misled by the dead, disturbed by gas leaks, and become fatigued or injured. Compared with other search techniques, such as infrared cameras and geophones, radars can emit waves that penetrate through rubble layers. Using the Doppler effect, a radar can detect the chest movement of living persons when they breathe. Such radars are called bioradars. By equipping unmanned aerial systems (UAS) with bioradars, rescue teams can efficiently search large disaster areas, increasing the chances of locating survivors quickly [1,2,3].

Bioradars designed for medical and biometric applications transmit and receive radio frequency (RF) waves in the air. They can detect changes in vital signs with high accuracy by using high operation frequencies such as the popular 2.4 GHz, 5.8 GHz, and 24 GHz ISM bands [4,5]. In contrast, ground-penetrating bioradars for post-disaster search face numerous challenges due to the harsh and complex operating environment [6]. One of the challenges is the high signal attenuation when RF waves propagate through rubble layers. Additionally, reflections between different layers of rubble cause energy loss and reduce the signal-to-noise ratio. Moreover, disturbances such as walking by individuals or operating machines can significantly degrade the signal quality. Even small changes in body position, such as arm movement, can also cause discontinuity of the reflected signal. Last but crucial, each disaster site has a different material composition and distribution, making the calibration of bioradar measurements difficult or impossible.

Many research studies have primarily focused on accurately identifying the frequencies of a person’s breathing and heartbeat signals in a high-attenuation environment while disregarding the diversity of disaster sites and the impact of the disturbances in actual post-disaster operations [6,7,8]. Scientific publications in this field often use complex diagrams, such as Fourier transform (FFT) spectra and continuous wavelet transform (CWT) time-frequency distributions, to visualize signal processing results. However, these diagrams are not helpful for first responders who need concise information during rescue operations, where the primary objective is to determine whether a survivor has been detected, which is a binary classification problem.

**Figure 1:**
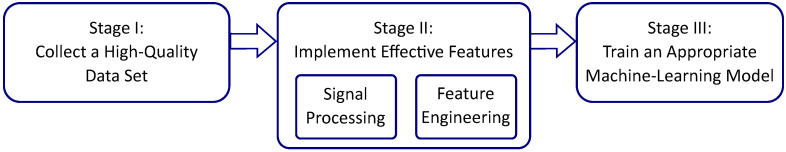
The three stages to design an automated life-detection algorithm.

The objective of this contribution is to design an automated life-detection algorithm that can overcome the challenges mentioned earlier. To achieve this objective, three stages are essential, as shown in Figure 1. First, it is necessary to collect a high-quality dataset by conducting systematic measurements in environments resembling post-disaster conditions. Second, the signals detected by the bioradar must be thoroughly examined to discover variables that can distinguish between measurements with and without the presence of a trapped person. These quantitative or qualitative variables, which result from measurements and are used to model the outcome, are known as features in machine learning terminology [9,10]. Finally, an appropriate machine learning model should be trained, taking into account the features’ characteristics and various model limitations.

The second stage, especially the feature engineering part, is the most time-consuming step in developing a machine-learning algorithm and often impacts the outcome quality more than the machine-learning model used [11]. Good features can express significant class differences. Models with too many features are less interpretable, computationally inefficient, and may suffer from overfitting [10,12]. The process of finding the optimal set of features that improve the model effectiveness is called feature engineering [10].

Which features are optimal depends largely on the application. There are many different classification applications using Doppler radar recorded breathing signals. Studies for different applications have chosen different features. Miao et al. used breath rate, short-time energy, and variance of short-time energy as features to classify normal breathing and three types of breathing disorders [13]. Ma et al. reported a method for distinguishing a standing human being from a dog in through-wall radar measurements. They have defined 12 features, including features corresponding to energy, time domain, and frequency domains [14]. Zhang et al. extracted 63 features from radar-captured respiratory and heartbeat signals and retained the 26 most significant features to classify four emotions: happy, relaxed, sad, and afraid [15]. Lin et al. defined eight time-domain features from radar-captured heartbeat signals to train a user authentication system [16]. Rahman et al. extracted ten time-domain features from radar-captured respiratory signals for subject identification [17].

To our best knowledge, this contribution reports the first attempt at systematically engineering features for a ground-penetrating bioradar application. Additionally, it is likely the first work that generates multiple virtual scenes from a single real scene, exploiting multipath propagation to expand the dataset’s size. To further enhance our understanding, we also conducted an investigation into how various factors, such as different individuals, body positions, slight movements, and signal strengths, affect the features.

For the convenience of reading, we denote all features we study in non-italic font and all other variables in italic font.

## 2. Measuring Principle and Method Overview

### 2.1. Bioradar Measuring Principle

When we breathe, our chest wall moves rhythmically. The chest displacement can be approximated by a sine function $x\left(t\right)=x_{\max}\sin\left(2\pi f_{\mathrm{resp}}t\right)$, where $f_{\mathrm{resp}}$ is the respiratory rate. The velocity v(t) of the chest motion is the derivative of x(t) to time:

(1) $$v\left(t\right)=\frac{dx(t)}{dt}=2\pi f_{\mathrm{resp}}x_{\max}\cos\left(2\pi f_{\mathrm{resp}}t\right).$$

This periodic velocity variation results in a periodically changing Doppler frequency $f_{D}$ in the reflected radar signal [18]:

(2) $$f_{D}=\frac{d\phi (t)}{2\pi dt}=\frac{2}{\lambda }v\left(t\right)=\frac{4\pi }{\lambda }f_{\mathrm{resp}}x_{\max}\cos\left(2\pi f_{\mathrm{resp}}t\right).$$

Throughout a one-minute measurement, the respiratory rate $f_{\mathrm{resp}}$ remains relatively stable, while the value and sign of the Doppler frequency $f_{D}$ exhibit periodic changes.

**Figure 2:**
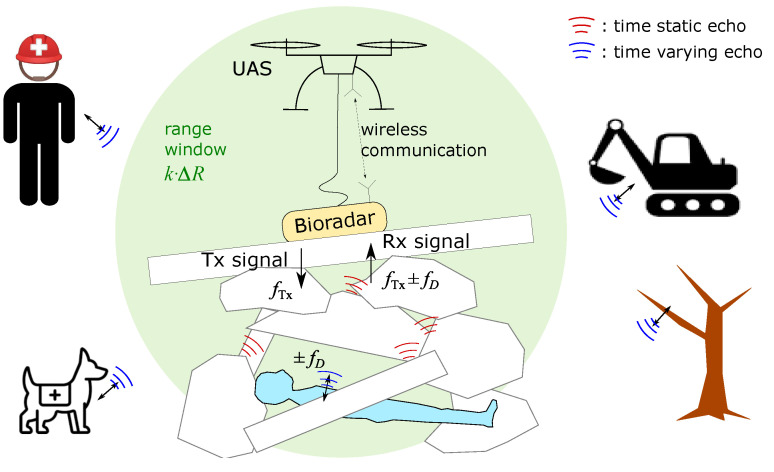
Illustration of a bioradar operation scenario. A person is trapped underneath rubble piles. A bioradar is placed by an UAS on top of the rubble pile. All objects in the environment reflect the transmitted radar signal. A first responder, a rescue dog, a working excavator and a vibrating tree are out of the range window.

Figure 2 depicts the complex operational environment of a rescue bioradar. There are usually many moving entities in disaster areas, such as first responders, rescue dogs, and machines. They may interfere with the bioradar measurements. However, by employing a range window, we can separate the signal of the trapped individual from interfering signals that occur at different distance intervals. The range resolution ΔR depends on the bandwidth BW of the applied radar signal and the propagation velocity *c* of electromagnetic (EM) waves in medi:.

(3) $$\Delta R=\frac{c}{2\;\cdot \;BW}.$$

Due to the unknown composition of the material and cavities beneath the rubble surface, it is impossible to obtain an accurate value for the actual *c*, and therefore the value of ΔR is hard to determine. However, it is certain that the range resolution ΔR will be smaller when measuring through the rubble than in the air.

### 2.2. Workflow and Outline of this Contribution

**Figure 3:**
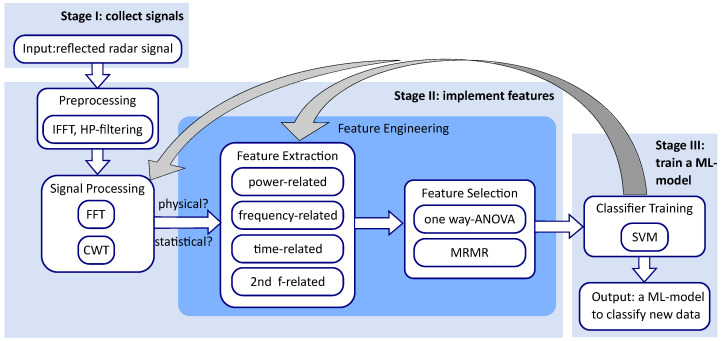
The workflow of this contribution, which is an expansion of the workflow given in Figure 1.

To achieve the automatic classifier, we developed a workflow to accomplish the three stages introduced in Figure 1, as depicted in Figure 3. During stage I, we will use the bioradar system developed in a previous study [8] to collect data by taking measurements in a laboratory experiment simulating a post-disaster scene. This experiment is further detailed in Section 3.

Stage II involves four steps. The first two steps, preprocessing of raw data and preliminary signal processing with Fourier transform (FFT) and continuous wavelet transform (CWT), were also introduced in detail in [8]. To bring readers into the topic of feature engineering, we will use a “with person” measurement as an example to introduce the signal processing routines in Section 3.2 briefly.

Feature engineering is the focus of the work. It involves two main steps: feature extraction and feature selection [19]. While feature selection can be made scientifically using mathematical methods, feature extraction is often a creative process requiring domain expertise and a deep understanding of the data [10].

Although the extraction is a creative process, some directions can guide us to extract candidate features more systematically. In the case of radar sensor signals, features can be divided into physical and statistical features. For time-varying signals, features can be derived from time-domain, frequency-domain, and time-varying-frequency representations [19]. Furthermore, according to their associated physical quantities, they can be categorized into time-related, frequency-related, and power-related features. In Section 4, the extraction of candidate features using these categories will be introduced in detail.

Feature selection is a process in machine learning where a subset of relevant features is selected from a larger set of extracted features. This is important because it can improve the accuracy and efficiency of machine learning models. Two commonly used methods for feature selection are one-way analysis of variance (ANOVA) [20] and minimum redundancy and maximum relevance (MRMR) [21]. In Section 5, we employ these methods to select important features. We will also explain the basics of one-way ANOVA and MRMR and demonstrate how they can be used to rank the extracted features. We select an optimal set of four features based on their rankings from the one-way ANOVA and MRMR methods, as well as their cross-validation accuracies when used in different combinations.

In the third stage, the selected features will be used to train a machine-learning model that can accurately classify the measurements. Given the complex measuring conditions of bioradar and the continuous nature of the features we have studied, certain machine-learning models are better suited for our problem than others. Nearest neighbor models and logistic regression are not suitable for our problem due to their sensitivity to outliers and requirement for linearly separable data, respectively [9,10].

Several models without these constraints can be applied to our problem. The accuracy and performance of a classifier are heavily influenced by the features used, so the focus of this contribution is primarily on feature engineering. We ultimately chose to use SVM due to its robustness and ability to produce easily interpretable decision boundaries [22,23], which is particularly useful for a problem with complex measuring conditions and continuous features. In Section 6, the results of this work are presented, including the hyperplane functions of the trained SVM models and the effect of signal prominence ratio and test person’s body position on the detection accuracy.

## 3. Data Collection

### 3.1. Experiment

**Figure 4:**
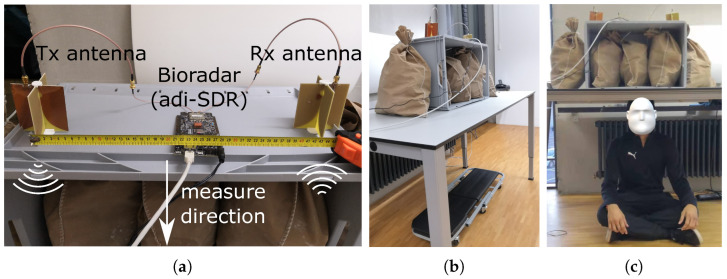
The experimental set-up. In the center is an office table with five bags of broken bricks, three bags are in a plastic box, and two are on the side. Under the table, there are two metal beams. (**a**) A bioradar and two antennas are on the top of the box. (**b**) Side view of the set-up. A test person can hide underneath the table. One long side of the set-up is close to a wall with a heater. (**c**) Body position 1: sitting.

To collect measurement data for training the classification model, we built an experimental set-up in our laboratory to simulate a building ruin with cavities. We used the frequency comb continuous wave (FCCW) bioradar developed in [8]. The bioradar sends and receives a FCCW signal with a bandwidth of about 60 MHz in the 1.3 GHz band. Figure 4 shows the construction of the experimental set-up. The wall opposite the set-up is four meters away, and there is a corridor outside where people occasionally walk by.

**Figure 5:**
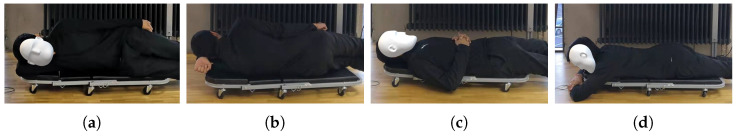
(**a**) Body position 2: right lateral. (**b**) Body position 3: left lateral. (**c**) Body position 4: supine. (**d**) Body position 5: prone.

In order to obtain robust features, the dataset needs to have sufficient diversity. Furthermore, the number of measurements shall be much higher than the number of features to reduce overfitting and enable cross-validation. For this contribution, we have taken 207 measurements of 20 test persons with the set-up. The test persons include 14 males and 6 females aged 20 to 65. In actual collapsed buildings, victims can be trapped in any body position. We are interested in whether people in certain body positions are easier to detect than others. Therefore, we have instructed the testers to adopt a different body position in each test. Five different body positions are investigated: sitting, right lateral, left lateral, supine, and prone, as illustrated in Figure 4c and Figure 5a–d. About 20% of the measurements are in a sitting position. The left and right lateral positions have 10%, respectively. The supine position takes the most measurements, with about 40% of all measurements. The rest 20% are in the prone position. The number of measurements across these positions is not the same because we guess most victims are in a lying-down body position when trapped. People in the supine position face the radar and can breathe most relaxedly. Furthermore, we expect the left and right lateral positions to have similar detection success rates. To balance the dataset, 226 control measurements without a test person are conducted with the same set-up.

### 3.2. Obtaining Multiple Virtual Scenes Using Multipath-Reflections

**Figure 6:**
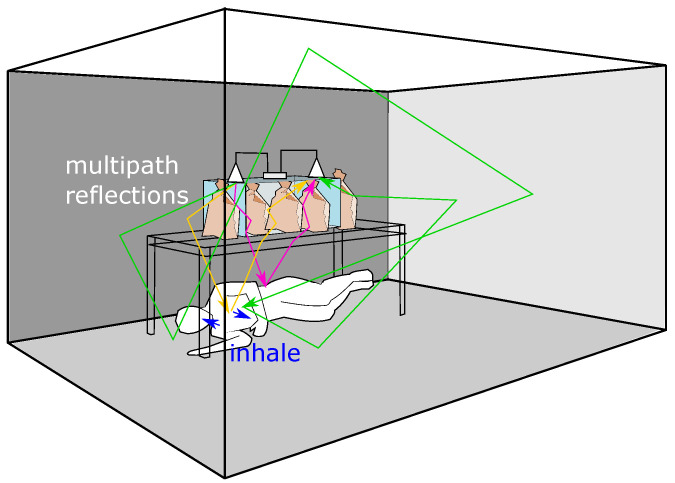
Schematic diagram of the laboratory with experimental set-up. A test person lies in a “right lateral” position. The blue arrows illustrate the deformation of the human body during inhalation. The transmission and reflection of RF waves can take any path in the room. Here we illustrate some possible paths with yellow, rose-red, and green colors. In the illustration, the rose-red colored path is the shortest, RF waves propagate through the box with brick bags and the table, hit the person’s waist then return. The green path is the longest, however, the waves in this path mainly travel through the air, reaching and reflecting perpendicular to the person’s chest.

Multipath-reflection is unavoidable for ground-penetrating bioradar applications because every interface in the rubble reflects radar waves. Depending on the scenario’s construction and the trapped survivor’s body position, the strongest signal may take a longer transmission and reflection path to the person than the fastest propagation path. As illustrated in Figure 6, even though the green path is longer than the rose-red path, its returned signal may be stronger due to less attenuation.

Although multipath propagation can cause signal interference and distortion and is annoying in many applications, we can exploit it to derive multiple virtual scenes from the real one. As described in [8], by using an inverse Fourier transform (IFFT), we can decompose the received frequency comb into different range intervals.

**Figure 7:**
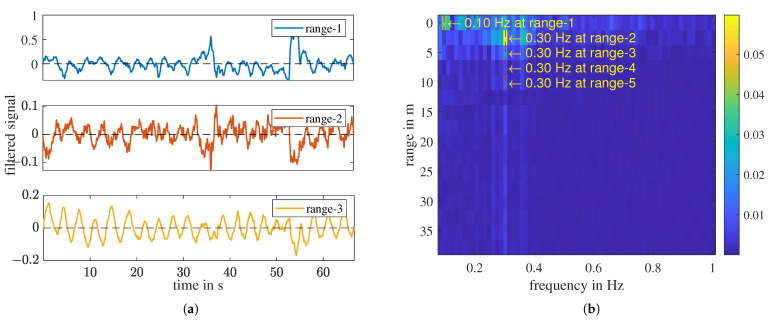
A measurement of “with person”. (**a**) Sub-signals of the first three range intervals, high-pass filtered. (**b**) Frequency—range plot. In the range 1 to 5, the FFT-determined respiratory frequency $f_{\mathrm{fft}}$ is labeled.

Figure 7a shows the first three sub-signals of a “with person” measurement. These sub-signals have different strengths but exhibit the same periodicity. Each range interval can be considered as a different test scenario. Each horizontal color strip in Figure 7b is the Fourier transform of the sub-signal in the corresponding range interval. We denote the frequency with the maximum magnitude in the frequency range [0.08, 1] Hz as the FFT-determined respiratory frequency $f_{\mathrm{fft}}$.

**Figure 8:**
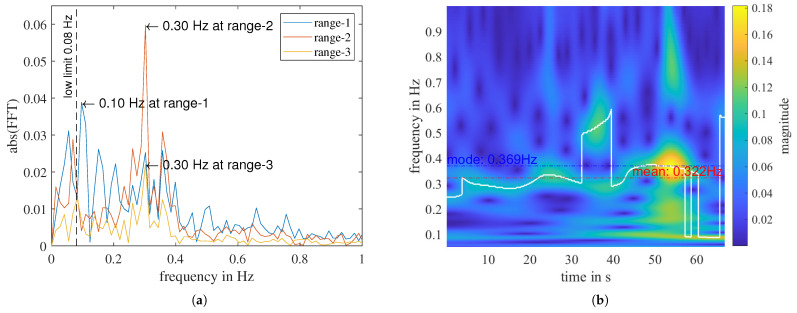
A measurement of “with person”. (**a**) FFT of the high-pass filtered signal of the first three range bins. (**b**) CWT of range 1. The biggest peak at each time point is highlighted with the white curve, $f_{\mathrm{cwt}}$. The mode and mean of $f_{\mathrm{cwt}}$ is noted on the plot with a dotted line, respectively.

**Figure 9:**
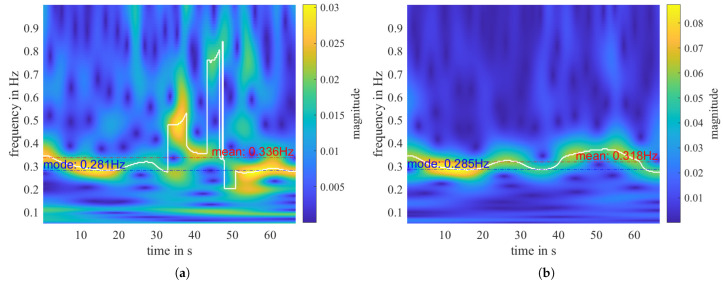
The “with person” measurement shown in Figure 8. (**a**) CWT of range 2. (**b**) CWT of range 3. The biggest peak f_cwt at each time point is highlighted with the white curve, $f_{\mathrm{cwt}}$. The mode and mean of $f_{\mathrm{cwt}}$ is noted on the plot with a dotted line, respectively.

In this measurement, for range-2 to range-5, $f_{\mathrm{fft}}$ is detected at 0.3 Hz. However, in range-1, it is detected at 0.1 Hz due to a low-frequency remainder despite the applied high-pass filter, which can also be seen in the FFT spectrum shown in Figure 8a. Apart from the static remainder, signal discontinuities can cause FFT distortion, leading to inaccurate determination of the respiratory frequency. These signal disruptions may arise from factors such as temporary breath-holding or body movement. To overcome this challenge, we utilize a second transform, the continuous wavelet transform (CWT), which enables us to track quasi-periodic components over time closely. The time-frequency distributions (TFD) of the CWT of the three sub-signals shown in Figure 7a are given in Figure 8b and Figure 9a,b, respectively.

To estimate the respiratory rate from CWT, we first define a measure called peak factor PF[n] for time point *n* in the TFD. The detailed definition can be found in [8]. The strongest peak at *n* has the largest PF[n], which is $PF_{\max}\left[n\right]$. The frequency value of these peaks, $f(PF_{\max}\left[n\right])$, builds a 1D-curve, as illustrated as the white curve in the TFD. In the following, we denote this curve by $f_{\mathrm{cwt}}$.

(4) $$f_{\mathrm{cwt}}=f\left(PF_{\max}\left[n\right]\right).$$

Interestingly, $f_{\mathrm{cwt}}$ curves in range-1 and range-2 sub-signals have jump discontinuities in the middle of the measurement but not in range-3. If we compare with Figure 7a, we can see that in the time domain, there are also small perturbations in the middle of the sub-signals of range-1 and range-2, but not in range-3. That indicates that the person probably moved a bit, but the movement is perpendicular to the arriving wave in range-3. Therefore, it has no impact on the sub-signal in range-3.

The frequency comb we use has 32 tones. Theoretically, we can use up to 32/2=16 sub-signals of one measurement to represent 16 measurements. For this contribution, we use the first five sub-signals. By doing so, we can construct a dataset with (207+226)·5=2165 measurements.

## 4. Feature Extraction

We start the feature extraction with the fundamental division: physical and statistical. Physical features are those variables meant to accurately characterize (describe) the signal of interest, which in the case of rescue bioradars is the breathing signal. Statistical features, in contrast, do not provide any direct physical description of the breathing signal. We first extract physical features from the transformations detailed in Section 3, and focus on analyzing signals in the frequency range of [0.08, 1] Hz.

### 4.1. Physical Features Related to Respiratory Rate

The estimated respiratory rate using FFT, denoted as f_fft, is a physical feature. We denote it now using non-italic font to emphasize its status as a candidate feature. The FFT spectrum exhibits its maximum magnitude $X_{\max}$ at the frequency f_fft. However, assuming that f_fft is the actual respiratory signal is incorrect. The reason is three-fold: first, the FFT spectrum always has a maximum value regardless of whether there is a victim; second, even if the signal does contain a breathing signal, due to interference, the breathing frequency may not be precisely the frequency with the largest magnitude; third, the human breathing rate is not strictly constant. Therefore, we cannot specify the signal, as well as the signal-to-noise ratio (SNR). Instead, we use signal prominence ratio (PR(f_fft)), a related metric that reflects the ratio of the signal power at f_fft to the average power of other points in the FFT magnitude spectrum X(k).

(5) $$\mathrm{PR}(\text{f\_fft})=\frac{X_{\max}^{2}}{(\sum_{k=1}^{L}|X\left(k\right){|}^{2}-X_{\max}^{2})/\left(L-1\right)},$$

where *k* is the index of discrete frequency in [0.08, 1] Hz and *L* is the number of points in this frequency range.

We can also estimate the respiratory rate from the time-frequency distribution obtained using CWT. The mode value of the curve $f_{\mathrm{cwt}}$ (Equation (4)) represents the dominant frequency of the signal and is a relatively robust and accurate parameter to describe the person’s respiratory rate during a measurement.

(6) $$\text{f\_cwt\_mode}=\text{mode}(f_{\mathrm{cwt}}).$$

Figure 9 shows that, even with perturbations in between, f_cwt_mode in range-2 is very close to f_cwt_mode in range-3. In contrast to f_cwt_mode, all disturbances and noise will affect the mean value of the $f_{\mathrm{cwt}}$ curve. The duration of the f_cwt_mode within a measurement, TD_cwt_mode, relates to the stability of the measured breath and can describe the quality of the respiration detection. Considering the inconstant nature of human breath, we define TD_cwt_mode as the duration of $f_{\mathrm{cwt}}$ in the frequency interval f_cwt_mode ± 0.05 Hz, normalized by the total measuring time $T_{\mathrm{meas}}$.

(7) $$\text{TD\_cwt\_mode}=\frac{T(f_{\mathrm{cwt}}=\text{f\_cwt\_mode}\pm 0.05\;Hz)}{T_{\mathrm{meas}}}\;\cdot \;100\%.$$

We chose a fixed interval width of 0.1 Hz instead of a floating value dependent on the f_cwt_mode because the average standard deviation of $f_{\mathrm{cwt}}$ for “with person” is approximately half of that for “without person”. In our later analysis, we will demonstrate that the mean standard deviation of $f_{\mathrm{cwt}}$ for “with person” measurements is 0.09 Hz, which is approximately equal to the interval width of 0.1 Hz, whereas for “without person” measurements, it is 0.19 Hz. Consequently, we can expect that with an interval width of 0.1 Hz, the TD_cwt_mode would exhibit statistically significant differences between the two classes of measurements.

### 4.2. Statistical Features

While statistical features may not have a direct physical interpretation of the breathing signal, they can help distinguish between signals with and without a person. For example, the standard deviation of $f_{\mathrm{cwt}}$, denoted with std(f_cwt), can describe the signal’s fluctuation from its mean [24]. A low value of std(f_cwt) indicates that the signal’s energy is concentrated over the measurement period, which could suggest the presence of a person in the rubble. Statistical features like std(f_cwt) are helpful because they can detect patterns in the signal that may not be visible through physical features alone.

**Figure 10:**
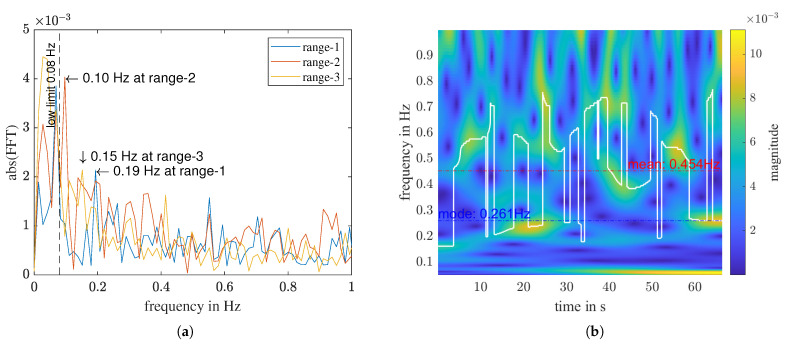
A “without person” measurement. (**a**) FFT of the high-pass filtered signal of the first three range bins. (**b**) CWT of range 1.

An example of the misleading nature of relying solely on extracted physical features can be seen in a “without person” measurement. Figure 10 shows that, for the first three range bins, f_fft has been determined as different values, ranging from 0.1 to 0.19 Hz, while the f_cwt_mode of range-1 is determined as 0.261 Hz. Although these frequency values fall within the breathing rate range of a normal relaxed human being, they do not represent a true breathing signal. In a measurement that genuinely captures breathing, the respiratory frequencies determined with different transforms should have relatively similar values. To address this issue, we define a normalized difference between f_fft and f_cwt_mode:

(8) $$\Delta f(\mathrm{fft},\;\text{cwt\_mode}=\frac{\left|\text{f\_fft}-\text{f\_cwt\_mode}\right|}{(\text{f\_fft}+\text{f\_cwt\_mode})/2}\;\cdot \;100\%.$$

It is a statistical feature that is derived from two frequency-related physical features. Another statistical feature is the mean value of the $f_{\mathrm{cwt}}$ curve:

(9) $$\text{f\_cwt\_mean}=\text{mean}(f\left(PF_{\max}\left[n\right]\right)).$$

In contrast to f_cwt_mode (Equation (6)), f_cwt_mean is affected by all noise and disturbances present in the measurement. Since we are investigating the frequency range of [0.08, 1] Hz, and most people breathe at a rate slower than 0.4 Hz, for a “with person” measurement, f_cwt_mean tends to have a higher value than f_cwt_mode, as shown in Figure 9. Conversely, for a “without person” measurement, where only noise of different kinds is present, f_cwt_mean tends to be close to the middle of the analyzed frequency range. For instance, in the signal illustrated in Figure 10, the f_cwt_mean is 0.454 Hz. Analogous to the mode-value-related features, we can define the corresponding TD_cwt_mean and Δf(fft, cwt_mean).

### 4.3. Summary and Analysis of Extracted Features

Table 1 lists the nine features that we have defined along with their mean μ and standard deviation σ for all “with person” and “without person" observations. The type column denotes the physical (phy.) or statistical (stat.) nature of the feature, as well as whether it is derived from the frequency domain (FD) or time-frequency domain (TFD). Furthermore, P, F, T, and F2 stand for power-related, frequency-related, time-related, and secondary frequency-related features, respectively. We have grouped these features into four categories based on their associated physical quantity type (P, F, T, or F2). Later, we will select one feature from each category to reduce redundancy.

**Figure 11:**
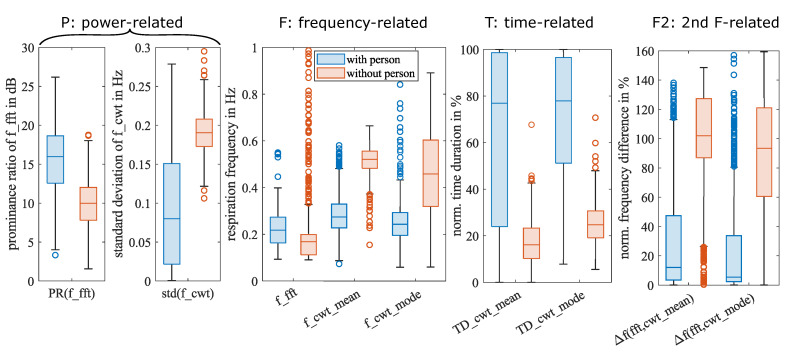
Boxplots of nine features for observations with and without persons, respectively. The median of a feature is shown as the line inside the box. The lower and upper quartiles are shown as the bottom and top edges of the box, respectively. The distance between the top and bottom edges is the interquartile range (IQR). Outliers are shown as circles, and they are values that are more than 1.5·IQR away from the edges of the box. The whiskers are lines that connect the box edges to the nonoutlier maximum and the nonoutlier minimum.

The mean and standard deviation are the most basic statistical measures. For a numeric dataset with a Gaussian distribution, they are sufficient. The features we selected, however, have unknown distributions. A box plot using median and quartiles can graphically display any distribution’s concentration, skewness, and outliers. Figure 11 shows the boxplots of the nine selected features for observations with and without persons. Features of the same category are listed together.

The box in the box plot represents the interquartile range, which includes 50% of the samples. The distance between the boxes representing “with person” and “without person” indicates the classification potential of each feature. A larger distance between the boxes suggests a stronger potential for classification. For instance, the boxes for f_cwt_mean are further apart than those for f_cwt_mode, which implies that f_cwt_mean is a more significant feature than f_cwt_mode.

All features, except for f_fft, have non-overlapping boxes, indicating a clear distinction between the two classes based on these features. The lack of separation in f_fft’s boxes suggests that it has limited classification potential. It is important to note that the high-pass filter used in the preprocessing stage has a cut-off frequency of 0.04 Hz, which can leave a static offset residue in the spectrum, as a result, in “without person” measurements, the lower edge of our frequency range of interest, 0.08 Hz, is often detected as f_fft.

## 5. Feature Selection

The boxplots give us an intuitive overview about the distributions of the candidate features. However, to select the optimal feature set, we need some numeric methods to calculate the significance of each feature and compare them. In this section, we investigate the significance of the nine extracted candidate features using two feature ranking methods. One is a univariate method: Analysis of Variance (ANOVA), and one is a multivariate method: Minimum Redundancy Maximum Relevance (MRMR). Based on the ranking of the features and the classification accuracy of different feature combinations, we can select an optimal feature set with minimal size.

### 5.1. Basics of One-Way ANOVA

ANOVA is widely used for hypothesis testing [25]. In feature engineering, the one-way ANOVA is utilized to analyze the significance of features individually by comparing the within-group and between-group variances of one feature. The null hypothesis $H_{0}$ states that the sample means of different classes do not differ significantly [20]. ANOVA utilizes the *F*-test statistic, which is the ratio of the mean square between groups (MSG) to the mean square error (MSE) [25]:

(10) $$F=\frac{MSG}{MSE}=\frac{SSG/d_{fG}}{\left(SST-SSG\right)/d_{fE}}.$$

The larger the *F*, the more substantial the evidence against the $H_{0}$. The one-way ANOVA tests one feature at each time. MSG and MSE are obtained by dividing the sum of squares between groups (SSG) and the sum of squared errors (SSE) by their respective degrees of freedom $d_{fG}$ and $d_{fE}$. SSE is the difference between the total sum of squares (SST) and SSG. For a binary classification problem with $N_{0}$ observations of group 0 and $N_{1}$ observations of group 1, the total sum of squares SST and the sum of squares between groups SSG of a feature *v* are defined as [25]:

(11) $$SST=\sum_{i=1}^{N_{0}+N_{1}}{\left(v_{i}-\;\bar{\;v\;}\;\right)}^{2},$$

(12) $$SSG=N_{0}{\left(\;\bar{\;v_{0}\;}\;-\;\bar{\;v\;}\;\right)}^{2}+N_{1}{\left(\;\bar{\;v_{1}\;}\;-\;\bar{\;v\;}\;\right)}^{2},$$

where $\;\bar{\;v_{0}\;}\;$ and $\;\bar{\;v_{1}\;}\;$ are the means of group 0 and group 1 are, respectively. $\;\bar{\;v\;}\;$ is the overall mean of the dataset. As there are only two groups for binary classification, $d_{fG}=2-1=1$ and $d_{fE}=N_{0}+N_{1}-2$ [25].

**Figure 12:**
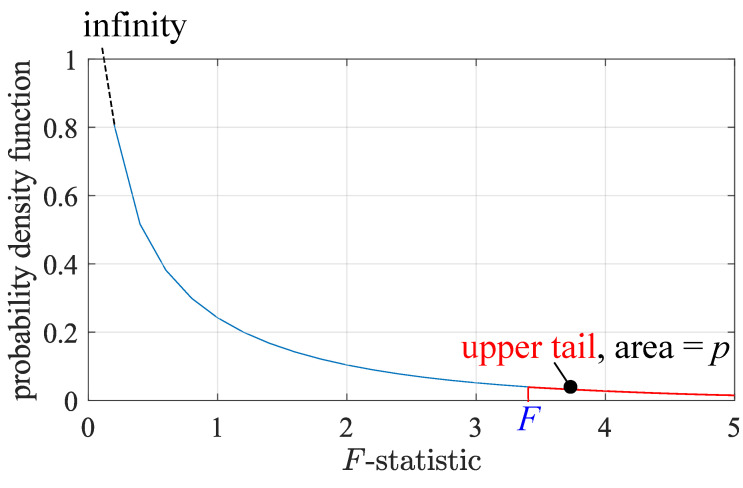
A *F*-distribution with $d_{fG}=1$ and $d_{fE}=2163$. The blue *F* is an illustrative example. The *p*-value is the area of the upper tail.

An *F*-distribution is defined with the two degrees of freedoms: $d_{fG}$ and $d_{fE}$ [25]. Figure 12 shows the *F*-distribution for our data, with $d_{fG}=1$ and $d_{fE}=2163$. The blue *F* here is an illustrative example. The *F*-statistics of our nine candidate features are all much greater than 5.

The area of the right hand side region of the *F*-statistic, the so-called upper-tail, is the *p*-value, which describes the probability that $H_{0}$ is true. The lower the *p*-value, the more critical the feature [25]. In Matlab, the one-way ANOVA scores a feature by its −ln(p).

### 5.2. Basics of MRMR

The Minimum Redundancy Maximum Relevance (MRMR) Algorithm [21,26] is based on the computation of mutual information. Two variables, *u* and *v*, can both be features, or one is a feature, and the other is the response. p(u) and p(v) are their probability distributions, respectively. p(u,v) is their joint probability distribution. The mutual information *I* of *u* and *v* is defined as the relative entropy from the product p(u)p(v) to p(u,v)

(13) $$I\left(u,v\right)=D_{\mathrm{KL}}\left(p\left(u,v\right)||p\left(u\right)p\left(v\right)\right)=\sum_{i,j}p\left(u_{i},v_{j}\right)\log\frac{p(u_{i},v_{j})}{p\left(u_{i}\right)p\left(v_{j}\right)},$$

where $D_{\mathrm{KL}}$ represents the operator of relative entropy, also known as the Kullback–Leibler divergence. Here we use the fscmrmr function in Matlab R2022a, which estimates the mutual information *I* for each pair of variables using an adaptive algorithm [26]. Suppose we have a feature set *S* with *M* features. For feature *u*, its redundancy $W_{u}$ is defined as the average of *I* between *u* and every other feature *z* in *S* and its relevance $V_{u}$ is the *I* between *u* and the response *y*:

(14) $$W_{u}=\frac{1}{M}\sum_{z\in S}I\left(u,z\right),$$

(15) $$V_{u}=I\left(u,y\right).$$

The MRMR score of a feature *u* is given by the quotient of its $V_{u}$ and $W_{u}$, the mutual information quotient MIQ:

(16) $$MIQ_{u}=V_{u}/W_{u}.$$

### 5.3. Ranking Results of Feature Importance

One-way ANOVA scores each feature independently. MRMR scores each feature depending on all other features. Table 2 shows the rankings and scores for the nine features given by one-way ANOVA and MRMR. In both rankings, f_fft has the lowest score, which matches our observation from the box plots in Figure 11.

**Figure 13:**
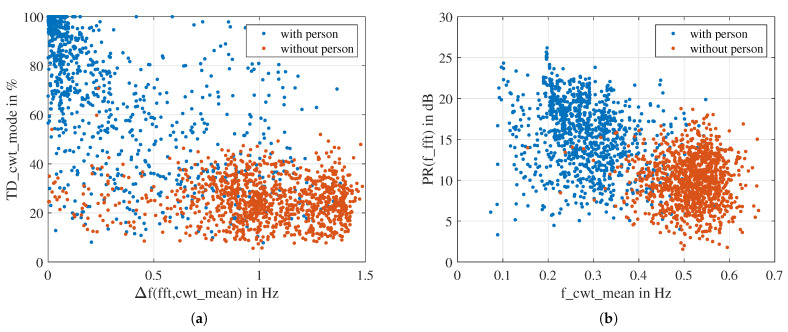
Scatter plot of the two top scoring features: (**a**) according to ANOVA ranking. (**b**) according to MRMR ranking.

Figure 13 shows a scatterplot of the two top-scoring features according to one-way ANOVA and MRMR, respectively. Each scatter represents an observation. We can see that in the two scatterplots, the observations of “with person” and “without person” are roughly divided into two clusters with some overlap. It is impossible to separate the two clusters with a straight line or a polynomial curve.

### 5.4. Investigate the Classification Accuracy of the Trained Models

To select an optimal feature set with the least number, we train support vector machine (SVM) models with augmented features according to the two rankings and analyze the accuracy and false rates of these models. To ensure reliable results, we utilize a five-fold cross-validation approach by randomly splitting the dataset into equal-size folds. Our analysis is focused solely on SVM models with linear kernels.

In the context of bioradar applications, we define “with person” as positive detection and “without person” as negative detection. False predictions can occur in two ways: when a “with person” measurement is classified as negative: false negative; or when a “without person” measurement is classified as positive: false positive. We do not discuss which of these two types of errors is more severe and set the misclassification cost to be equal for both cases.

**Figure 14:**
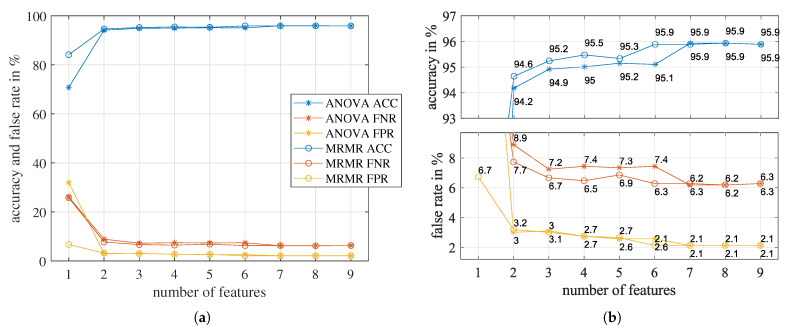
The classification accuracies (ACC), false negative rates (FNR), and false positive rates (FPR) of the SVM model as the number of features increase. Features are added sequentially according to the rank orders given by one-way ANOVA and MRMR, respectively. (**a**) overview (**b**) local zoom-in of (**a**).

Figure 14a shows the classification accuracies (ACC), false negative rates (FNR), and false positive rates (FPR) of the trained SVM models. Figure 14b zooms in on the accuracies and false rates, respectively. For both rankings, the classification accuracy improved significantly by increasing the number of features from one to three. After three features, for MRMR ranking, the most significant improvement of 0.6% occurs when the number of features is increased from five to six, which indicates the 6th feature is essential. For the one-way ANOVA ranking, the most significant improvement of 0.7% occurs at the 7th feature.

However, increasing the number of features did not constantly improve the prediction accuracy of the SVM model. From Figure 14, we can see that the accuracy has no improvement for more than seven features, indicating that redundant features exist. Furthermore, for models using more than one feature, FNR is always higher than FPR. By increasing the misclassification cost for FNR, the FNR will decrease, but the FPR will increase, and the overall prediction accuracy will reduce.

Comparing Table 2, we can see that the [1, 2, 3, 7] feature set of ANOVA has exactly the same features as the [1, 2, 3, 6] feature set of MRMR. They are [PR(f_fft), f_cwt_mean, TD_cwt_mode, Δf(fft, cwt_mean)]. Coincidentally, they are the assembly of the two highest-scoring features in each ranking. We decided to use this four-feature set. Since they also belong to the four different categories in Table 1, the redundancy of this set is minimal. The accuracy of the model trained with this feature set is 95.7%.

To analyze if we have collected enough measurements for feature engineering, we want to investigate whether we will obtain the same optimal feature set with a smaller dataset. To do so, we randomly divided the 433 measurements dataset into five groups, four groups with 87 measurements each and one with 85 measurements, so each group contains about 20% of the total measurements. The division also maintained consistency in the proportion of the two classes, with each group having less than a 2% difference in the percentage of “with person” measurements from the overall ratio of 207/433 = 47.81%. By analyzing the ranking of the nine candidate features using only the first 20%-group, the top four features identified through MRMR are f_cwt_mean, Δf(fft, cwt_mode), TD_cwt_mode, and PR(f_fft). The ranking sequence differs from the results obtained using all measurements. However, when the analysis included the first two groups, namely 40% of the measurements, the ranking order of the top four features aligned exactly with the order obtained using the entire dataset. When the dataset for analysis expanded to 60% and 80%, the results remained unchanged. Notably, the top four features identified through ANOVA ranking for the 20% subset matched those obtained using the complete dataset. These results indicate that achieving the same selected feature-set outcome is possible using just 40% of our collected measurements. With 433 measurements, our dataset is more than sufficient for accurate analysis and feature selection.

## 6. Results and Discussions

### 6.1. Hyperplane of Solved SVM Model

The dimensionality of a model is determined by the number of features used. A solved binary SVM model has a decision boundary, the so-called hyperplane, that optimally separates the training data into two classes. The dimension of a hyperplane is the problem space’s dimension minus one [27]. Therefore, the hyperplanes for 1D, 2D, and 3D-SVM models are points, lines, and planes, respectively. In an SVM model that employs *M* features, each observation *x* is represented by an *M*-dimensional feature vector. When using a linear kernel in the SVM model, the score f(x) will be a linear function of the observation *x* [9]

(17) $$f\left(x\right)=\left(\frac{x-\mu }{\sigma }\right)\frac{\beta }{s}+b,$$

where μ and σ are the training dataset’s mean and standard deviation, respectively, β is the weight of the features in the set. μ, σ and β are all *M*-by-1 vectors. The kernel scale *s* and model bias *b* are scalar parameters. Regardless of the model dimension, the function of the hyperplane is

(18) f(x)=0.

The classification of a new data vector *x* is determined by the sign of its corresponding score f(x). Table 3 presents the parameters of the trained linear support vector machine (LSVM) models using two, three, or four features from the selected feature set.

**Figure 15:**
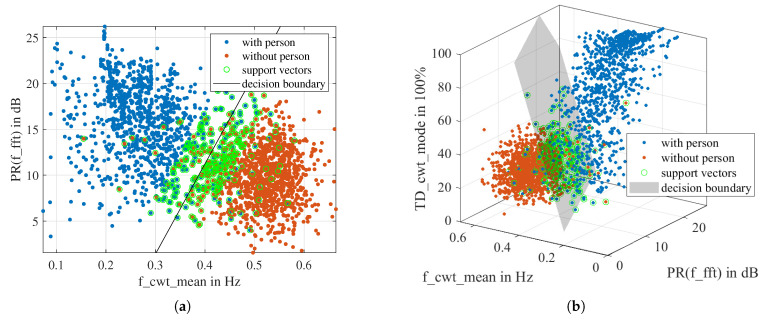
Scatter plots of the data and support vectors for the trained models. (**a**) The decision boundary of the 2D-LSVM model is a straight line. (**b**) The decision boundary of the 3D-LSVM model is a plane.

As the same training dataset is used for all models, the parameters μ and σ remain unchanged. However, because the dataset is randomly partitioned for training and testing, a trained model of the same dimension may have slight variations in the kernel scale *s*, bias *b*, and weight β. Figure 15 demonstrates the scatter plots and decision boundaries of the 2D-LSVM model and the 3D-LSVM model. It can be seen that the two classes of observations can be roughly separated by the hyperplane.

**Figure 16:**
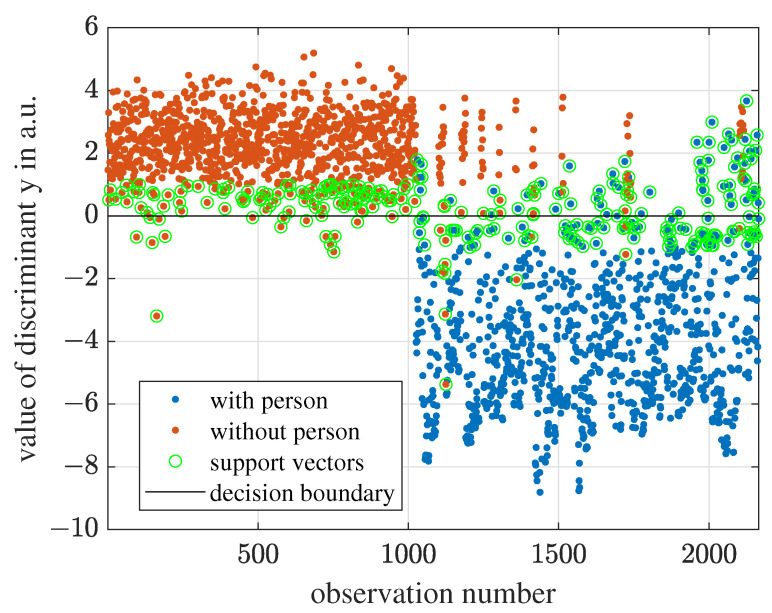
For 4D-LSVM, the observations can be wrapped into a 1D space according to the Equation (17). The decision boundary is the point f(x)=0.

For the 4D-LSVM model, we cannot visualize the 4D scatter plot and the 3D hyperplane. However, as illustrated in Figure 16, we can transform the 4D-feature space into a 1D-function as described in Equation (17), and the hyperplane is the point f(x)=0. The observations with f(x)<0 are classified as “with person” and the observations with f(x)>0 are classified as “without person”. The larger the magnitude of f(x), the more confident the prediction is.

Table 4 and Table 5 show the results of a “with person” measurement and a “without person” measurement, respectively. The predictions are made using the trained 4D-LSVM model. The five sub-signals of the “with person” measurement are all correctly classified. For the “without person” measurement, the second range sub-signal is misclassified, but it has the smallest |f(x)|.

### 6.2. Impact of Prominence Ratio on False Rate

**Figure 17:**
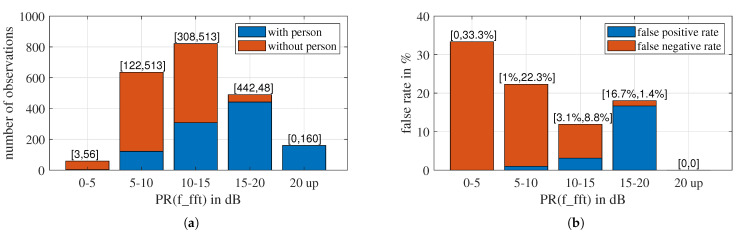
(**a**) The number of observations with different PR(f_fft) ranges. (**b**) The false rate of the 4D-LSVM model for different PR(f_fft) ranges.

Among the four features of the selected set, the signal prominence ratio PR(f_fft) is a special one. Like SNR, PR(f_fft) describes the prominence of a signal relative to the background and thus determines the credibility of other features. Here, we investigate the impact of PR(f_fft) on detection accuracy and question whether it will be possible to detect breathing when PR(f_fft) is small. We divide the observations into five groups according to their PR(f_fft) values and use different colors to mark the two classes, as illustrated in Figure 17a. The PR(f_fft) distribution for all observations is approximately Gaussian distributed. This is the same as what we learned from Figure 11; the majority of “with person” observations have higher PR(f_fft) than “without person” observations. Notably, there are only three “with person” observations for PR(f_fft) < 5 dB, which is less than the number of sub-signals of a measurement, five. Conversely, all observations with PR(f_fft) > 20 dB are “with person”.

Figure 17b shows the false rate of different PR(f_fft) groups. The lower the PR(f_fft), the higher the false negative rate (FNR). However, in this experiment, breathing is still detectable even for the lowest PR(f_fft) group. Out of the 59 observations in the PR(f_fft) < 5 dB group, only three are “with person”, but only one of those three was misclassified, resulting in an FNR of 33.3%. As PR(f_fft) increases, FNR reduces, and FPR grows. For PR(f_fft) > 20 dB, there is no “without person” observation, and all “with person” observations are correctly classified. Therefore, both FPR and FNR are zero.

### 6.3. Effect of Body Position on Breathing Detection in This Particular Experiment

**Figure 18:**
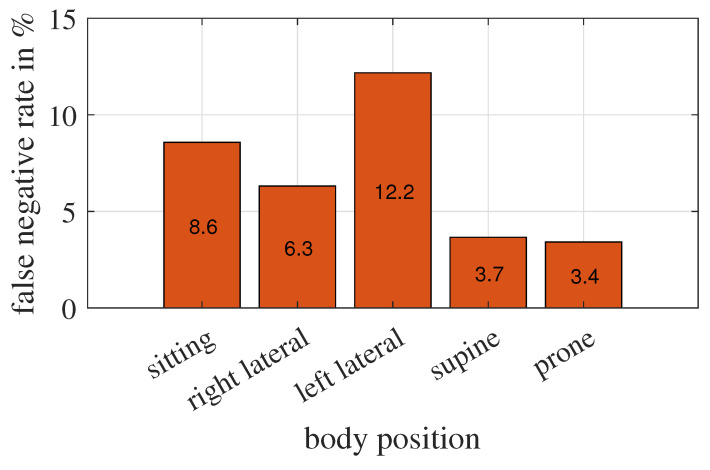
False-negative classified observations and corresponding body positions. The percentage of measurements taken in position sitting, right lateral, left lateral, supine, and prone are about 20%, 10%, 10%, 40%, and 20%, respectively.

Furthermore, we are interested in whether the body position of the test person affects the detection accuracy in this particular experiment. To investigate this question, we count the number of false negative classifications for each pose separately, as shown in Figure 18.

The radar measures downwards. In the supine and prone positions, the chest or back movement of the test person is axial to the radar, resulting in the most significant change in the radar cross-section. Measurements of these two body positions have also the lowest FNR. Similarly, we would expect that in the sitting position, the chest movement is perpendicular to the propagation direction of the radar wave, which causes a minimal change in the radar cross-section, therefore, is hardest to detect. However, observations of the left lateral position have the lowest accuracy. Their FNR is nearly doubled compared to the observations of the right lateral position, which seems strange but consistent with our discussion in Section 3.2. The experimental set-up is asymmetric and closer to the wall with the heater on the left side of the lying test person. The space on the right side is more open, and radar waves reach the person more easily from the right side than from the left.

From this study, we can say that the body position itself does not affect the detection accuracy, but rather the combination of the construction of the measuring scene and the body position of the trapped person does.

## 7. Conclusions

This contribution presents a systematic feature engineering approach for search and rescue bioradar applications. Through this process, we have identified an optimal feature set of four features related to four different physical quantities. These features possess consistent analytical meaning and the optimal feature set will stay significant and will not be influenced by the specific measurement scenario. This will efficiently enhance the classification’s reliability and accuracy.

Using a support vector machine (SVM), we trained a classifier model with an analytical decision boundary function that can be easily employed to classify new data automatically. The feature engineering process and visualized SVM hyperplanes also helped us better understand the application and bioradar signals.

While the complexity of rescue-bioradar measuring conditions makes it impossible to construct a comprehensive training dataset that covers all possible scenarios, our work represents the first attempt to achieve representative features despite this complexity. However, it is crucial to conduct further experiments close to actual post-disaster conditions, incorporating different building materials and constructions, to verify the significance of our selected feature set and enhance the robustness of the model. Additionally, for future iterations of training, expanding the dataset to include measurements of human infants and children will be essential. Only by continuing to refine and extend the dataset we can improve the detection success rate in practical urban search and rescue operations.

## Figures and Tables

**Table 1:** Nine extracted features.

#	Feature Notation	Type	Meaning	With Person		Without Person	
				μ	σ	μ	σ
1	PR(f_fft)	phy. FD-P	prominence ratio in dB	15.52 dB	4.26 dB	9.89 dB	2.96 dB
2	std(f_cwt)	stat. TFD-P	standard deviation of f_cwt	0.09 Hz	0.07 Hz	0.19 Hz	0.03 Hz
3	f_fft	phy. FD-F	frequency with max(FFT)	0.22 Hz	0.08 Hz	0.18 Hz	0.12 Hz
4	f_cwt_mean	stat. TFD-F	mean value of f_cwt	0.28 Hz	0.08 Hz	0.51 Hz	0.06 Hz
5	f_cwt_mode	phy. TFD-F	mode value of f_cwt	0.24 Hz	0.10 Hz	0.46 Hz	0.19 Hz
6	TD_cwt_mean	stat. TFD-T	norm. duration of f_cwt_mean	62.9%	37.1%	17.2%	9.4%
7	TD_cwt_mode	phy. TFD-T	norm. duration of f_cwt_mode	71.2%	26.8%	25.2%	8.7%
8	Δf(fft, cwt_mean)	stat. F2	norm. diff. btw. f_fft and f_cwt_mean	29.8%	35.2%	101.7%	30.2%
9	Δf(fft, cwt_mode)	stat. F2	norm. diff. btw. f_fft and f_cwt_mode	23.9%	34.1%	88.2%	41.7%

**Table 2:** Feature rankings and scores.

Ranking	One-Way ANOVA		MRMR	
	Feature	Score	Feature	Score
1	Δf(fft, cwt_mean)	Inf.	f_cwt_mean	0.51
2	TD_cwt_mode	Inf.	PR(f_fft)	0.42
3	f_cwt_mean	Inf.	TD_cwt_mode	0.36
4	std(f_cwt)	687.98	Δf(fft, cwt_mode)	0.34
5	TD_cwt_mean	604.74	std(f_cwt)	0.32
6	Δf(fft, cwt_mode)	580.65	Δf(fft, cwt_mean)	0.29
7	PR(f_fft)	510.40	f_cwt_mode	0.28
8	f_cwt_mode	467.40	TD_cwt_mean	0.24
9	f_fft	33.26	f_fft	0.12

**Table 3:** Parameters of SVM models trained with linear kernels with different numbers of features.

			2D-LSVM		3D-LSVM		4D-LSVM	
			*s*	*b*	*s*	*b*	*s*	*b*
			0.11	−0.17	0.25	−0.62	0.43	−0.69
**feature **	μ	σ	β		β		β	
PR(f_fft)	12.58	4.60	−0.09		−0.19		−0.31	
f_cwt_mean	0.40	0.14	0.25		0.50		0.79	
TD_cwt_mode	0.47	0.30	/		−0.26		−0.41	
Δf(fft, cwt_mean)	0.67	0.49	/		/		0.18	
**accuracy**	/		94.6%		95.2%		95.7%	

**Table 4:** Feature values of the “with person” measurement shown in Figure 8 and Figure 9. The predictions are made using the trained 4D-LSVM model.

	PR(f_fft)	f_cwt_mean	TD_cwt_mode	Δf(fft, cwt_mean)	f(x)	Prediction
range-1	13.39	0.32	0.37	1.08	−1.25	with person
range-2	17.83	0.34	0.70	0.11	−3.62	with person
range-3	16.28	0.32	0.69	0.05	−3.66	with person
range-4	14.54	0.32	0.74	0.05	−3.55	with person
range-5	17.25	0.40	0.13	0.28	−0.72	with person

**Table 5:** Feature values and predictions of the “without person” measurement shown in Figure 10.

	PR(f_fft)	f_cwt_mean	TD_cwt_mode	Δf(fft, cwt_mean)	f(x)	Prediction
range-1	10.20	0.45	0.23	0.81	1.24	without person
range-2	11.59	0.35	0.35	1.14	−0.46	with person
range-3	10.67	0.44	0.25	0.97	1.04	without person
range-4	11.35	0.55	0.26	0.81	2.27	without person
range-5	11.12	0.53	0.26	1.38	2.53	without person

## Data Availability

Data is available in the Zenodo repository, https://doi.org/10.5281/zenodo.7799440 (accessed on 1 June 2023).
